# Integrating multimodal information in machine learning for classifying acute myocardial infarction

**DOI:** 10.1088/1361-6579/acc77f

**Published:** 2023-04-18

**Authors:** Ran Xiao, Cheng Ding, Xiao Hu, Gari D Clifford, David W Wright, Amit J Shah, Salah Al-Zaiti, Jessica K Zègre-Hemsey

**Affiliations:** 1 School of Nursing, Emory University, United States of America; 2 Department of Biomedical Engineering, Georgia Institute of Technology & Emory University, United States of America; 3 Department of Biomedical Informatics, School of Medicine, Emory University, United States of America; 4 Department of Computer Science, College of Arts and Sciences, Emory University, United States of America; 5 Department of Emergency Medicine, School of Medicine, Emory University, United States of America; 6 Division of Cardiology, School of Medicine, Emory University, United States of America; 7 School of Nursing, University of Pittsburgh, United States of America; 8 School of Nursing, University of North Carolina at Chapel Hill, United States of America

**Keywords:** myocardial infarction, acute coronary syndrome, machine learning, electrocardiography, deep learning

## Abstract

*Objective*. Prompt identification and recognization of myocardial ischemia/infarction (MI) is the most important goal in the management of acute coronary syndrome. The 12-lead electrocardiogram (ECG) is widely used as the initial screening tool for patients with chest pain but its diagnostic accuracy remains limited. There is early evidence that machine learning (ML) algorithms applied to ECG waveforms can improve performance. Most studies are designed to classify MI from healthy controls and thus are limited due to the lack of consideration of ECG abnormalities from other cardiac conditions, leading to false positives. Moreover, clinical information beyond ECG has not yet been well leveraged in existing ML models. *Approach.* The present study considered downstream clinical implementation scenarios in the initial model design by dichotomizing study recordings from a public large-scale ECG dataset into a MI class and a non-MI class with the inclusion of MI-confounding conditions. Two experiments were conducted to systematically investigate the impact of two important factors entrained in the modeling process, including the duration of ECG, and the value of multimodal information for model training. A novel multimodal deep learning architecture was proposed to learn joint features from both ECG and patient demographics. *Main results.* The multimodal model achieved better performance than the ECG-only model, with a mean area under the receiver operating characteristic curve of 92.1% and a mean accuracy of 87.4%, which is on par with existing studies despite the increased task difficulty due to the new class definition. By investigation of model explainability, it revealed the contribution of patient information in model performance and clinical concordance of the model’s attention with existing clinical insights. *Significance.* The findings in this study help guide the development of ML solutions for prompt MI detection and move the models one step closer to real-world clinical applications.

## Introduction

Cardiovascular disease (CVD) remains the leading cause of death in the United States and around the world. Acute coronary syndrome (ACS) is the acute manifestation of myocardial ischemia/infarction (MI) and relies on rapid and accurate identification. Although nearly 6 million adults in the US are evaluated annually for chest pain, only a small subset are diagnosed with ACS (Al-Zaiti *et al* 2022a, 2022b). The most important goal in the management of ACS is identifying evidence of acute ischemia early and initiating prompt intervention to prevent adverse clinical outcomes (Farquharson *et al* 2019).

To date, the 12-lead electrocardiogram (ECG) remains the most widely used initial screening tool for evaluating patients with chest pain. Considered the ‘gold standard’ for non-invasive electrocardiographic detection of acute myocardial ischemia/injury, the standard 12-lead is noninvasive, inexpensive, and the single most important method for rapid detection of ACS in emergency care settings (i.e. prehospital, emergency department) (Goldman and Kirtane 2003, Zegre-Hemsey *et al* 2020). Current American heart association (AHA) guidelines recommend ECG acquisition within 10 min of first medical contact for individuals with symptoms suggestive of ACS including, but not limited to, non-traumatic chest pain, shortness of breath, radiating pain to arm or jaw, nausea, diaphoresis, or lightheadedness. The diagnostic accuracy of ECG for ACS by current threshold-based clinical rules, however, has only 30%–70% sensitivity and 70%–95% specificity (Fesmire *et al* 1998, Kudenchuk *et al* 1998). Approximately 50%–55% of patients with acute myocardial ischemia and 40% with unstable angina do not have ST-segment elevation or depression present in their initial ‘snap-shot’ ten-second 12-lead ECG recording (Forberg *et al* 2009, Rouan *et al* 1989). Continuous ECG monitoring has shown many ischemic events are clinically silent and transient and thus may not be captured by a single standard 12-lead ECG (Chantad *et al* 2006). Another challenge to current ECG is the visual interpretation of ST-segment deviation, which is exacerbated by high inter-rater variability, artifacts in ECG waveforms, and failure to detect subtle ischemic ECG changes. Each can compromise the reliability and validity of the ECG.

Machine learning (ML) has the potential to improve the diagnostic accuracy of MI because it is data-driven and not limited by preset rigid features and thresholds inherent to clinical rule-based algorithms. Many studies have recognized the opportunity and leveraged ML for classifying MI. Jager and colleagues implemented an unsupervised machine learning approach, the Karhunen—Loève (KL) transform, for the automatic detection of transient ST-segment episodes in continuous ambulatory ECG recordings, and differentiated them from non-ischemic ST events (Smrdel and Jager 2004, Jager *et al* 1998). A similar approach was later adopted for classifying subtypes of ischemic heart disease (Smrdel and Jager 2011). Wang *et al* combined statistical and entropy features extracted from ECG waveforms and implemented principal component analysis to reduce the dimension of features for training various shallow machine learning models to classify between MI and health control (Wang *et al* 2020). Similarly, Chang *et al* extracted sequential features in the temporal dynamics of ECG using a hidden Markov model (HMM) to train a support vector machine for the classification of MI and healthy control (Chang *et al* 2012). In another study, Sharma *et al* utilized multiscale wavelet coefficients as features for detecting MI (Sharma *et al* 2015). It is worth noting that the latter two studies did not consider patient assignments in the model development process (i.e. data from the same patient may exist in both training and test sets), which may artificially increase the model performance.

Inspired by promising achievements in the classification of cardiac arrhythmia, deep learning has gradually found its footing in studies for detecting MI. Reasat and Shahnaz used a convolutional neural network (CNN) and waveforms from three (i.e. lead II, III, and aVF) of the standard 12-lead ECG to classify between inferior myocardial infarction and healthy control (Reasat and Shahnaz 2017). Strodthoff *et al* conducted a comprehensive study comparing the performance of seven different deep-learning architectures in classifying five diagnostic classes available in the PTB-XL dataset (Wagner *et al* 2020, Strodthoff *et al* 2021).

While the above studies set the stage for leveraging machine learning to improve diagnostic accuracy for MI using ECG, several limitations exist in current knowledge. First, most of the existing studies formulate the classification problem as MI versus healthy control, which may overstate their performance and limits the clinical applications because many non-MI conditions can be present aside from the normal condition. Some of these conditions display electrocardiographic features confounding MI, such as left ventricular hypertrophy (LVH) and left bundle branch block (LBBB). Therefore, clinically viable algorithms should factor in these conditions. Second, existing studies use ECG of default length provided in the development dataset. It is still unclear about the impact of the duration of ECG on the performance of the MI detection model. Third, other types of information routinely collected (such as patient demographics, past diagnoses, and symptoms) during the initial evaluation of MI have not been well incorporated into the ML modeling process yet, despite existing evidence showing their diagnostic values in the initial evaluation phase for MI (Thygesen *et al* 2018).

The present study builds upon existing works (Chang *et al* 2012, Sharma *et al* 2015, Reasat and Shahnaz 2017, Wang *et al* 2020, Wagner *et al* 2020, Strodthoff *et al* 2021, Zegre-Hemsey *et al* 2021) and aims to advance ML-based classification models closer to clinical applications for the prompt detection of MI. We achieved this by considering downstream clinical implementation in our experimental design and dichotomizing recordings in the development dataset into MI and non-MI classes. We then conducted two separate experiments to systematically investigate the impact of two critical factors in the modeling process, the duration of ECG and the value of multimodal information entering model training. To this end, a novel feature-fusion deep learning architecture was proposed that can learn joint features from both ECG and patient demographics. The findings of the study have the potential to guide further work toward clinically oriented MI classification models with improved performance.

## Methods

### Study data

**Figure 1.**
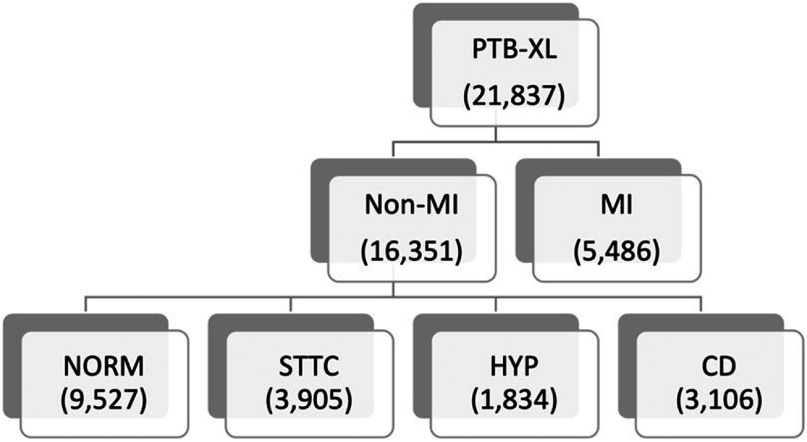
Breakdown of numbers of ECG recordings across different diagnostic statements.

We used a de-identified clinical ECG database for our study, the PTB-XL dataset, which is publicly accessible on the PhysioNet website (Goldberger *et al* 2000, Wagner *et al* 2020). The dataset includes 21 837 clinical 12-lead ECG records with durations of 10 s. The data were collected from 18 885 patients who are 52% male (48% female), with a median age of 62 and an interquartile range of 22. Baseline patient characteristics (i.e. age and sex) and clinical annotations are provided along with each ECG record. At least one cardiologist (up to two) annotated ECGs with three diagnostic categories, i.e. statement, form, and rhythm, in accordance with the SCP-ECG standard (International Organization for Standardization 2009). Based on the clinical annotations in the dataset, our study dichotomized ECG recordings into two classes, a MI class (5486 recordings) and a non-MI class (16 351 recordings). The non-MI class consists of recordings that are annotated as either normal ECG (NORM), or as any of the following three diagnostic statements, ST/T change (STTC), hypertrophy (HPY), and conduction disturbance (CD). The detailed breakdown of recordings across different diagnostic statements can be found in figure 1.

The dataset was prepared following a nine-fold cross-validation (CV) scheme, by first dividing it into ten equal partitions stratified across classes as provided by the PTB-XL dataset (Wagner *et al* 2020). The tenth fold served as the independent test set. The remaining nine folders were cycled nine times. Within each cycle, one different fold of data served as the validation set, and the remaining eight folds served as the training data. The data split considers underlying patient assignments by assigning recordings of the same patient to the same partition. This avoids any data leakage between training and test sets, which could artificially boost model performance if not considered. The institutional review board (IRB) of Emory University approved the study.

### Model development

**Figure 2.**
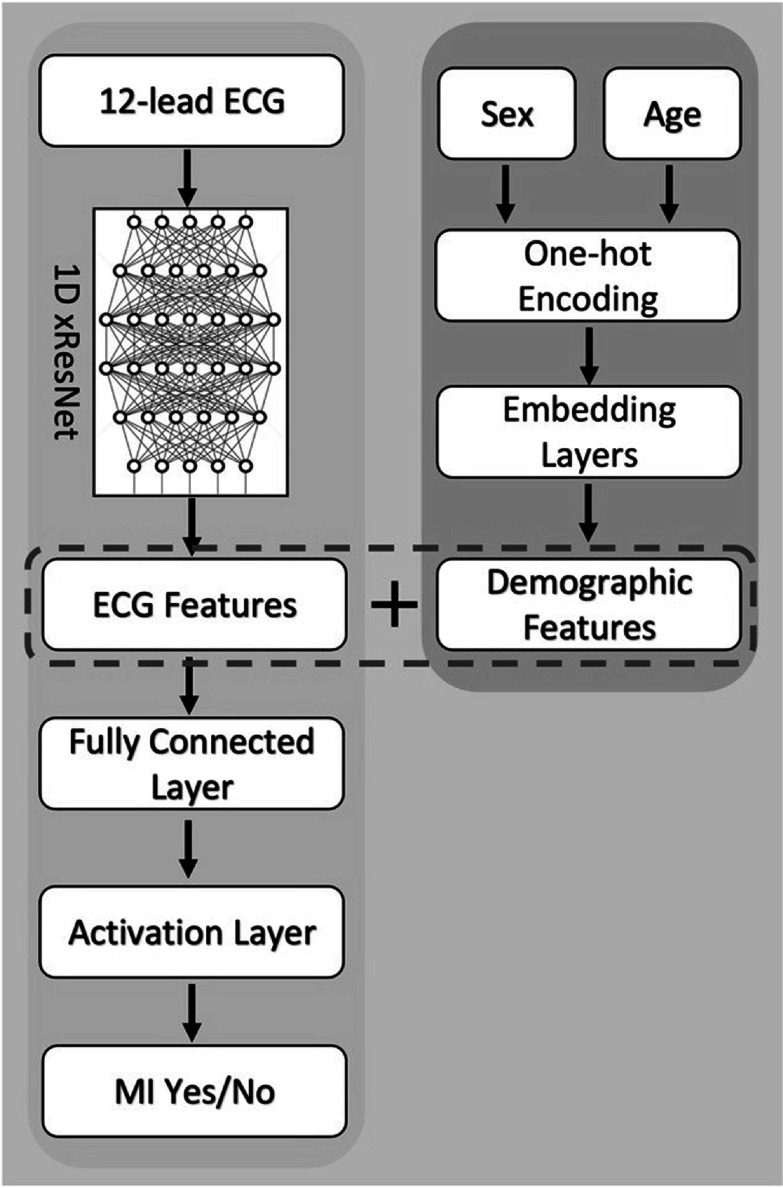
Schematic diagram of the ECG-only model (yellow) and the Multimodal model (yellow + blue).

We adopted two deep learning architectures that use different sources of inputs for the classification of myocardial infarction. The ECG-only model used only ECG data as the model input. The 12-lead ECG waveforms were represented as 12 channels of one-dimensional temporal signals to train a 1D CNN, xResNet (He *et al* 2018). xResNet is a variant of the ResNet deep learning model (He *et al* 2015), by building upon the ResNet architecture and implementing a collection of practical structural tweaks that were proven to improve the performance over the vanilla ResNet model. xResNet was selected in the study as it is one of the top-performing models among various deep learning models tested on the PTB-XL dataset (Strodthoff *et al* 2021). As shown in the yellow region of figure 2, 12-lead ECGs in the training set are input into the 1D xResNet model, which utilizes 101 convolutional layers to learn a comprehensive representation of ECG temporal dynamics, and outputs a classification for the diagnosis of myocardial infarction.

The second model architecture possesses a dedicated learner for patient demographic information (blue region, figure 2), and integrates it with ECG features learned from the xResNet model (yellow region, figure 2) to form the final Multimodal model. The demographic learner consisted of three main components. The first component used one-hot encoding to achieve a numerical representation of different demographic variables. For age and sex, binary vectors with lengths of 99 and 2 were used to represent the corresponding patient information. Next, an embedding layer was deployed to encode patient demographics into a continuous vector space that could enhance the representation of underlying information. Age and sex information were embedded in vectors with lengths of 16 and 128, respectively. Lastly, both age and sex were upscaled to 512-element vectors through 1D convolutional layers, which generated the final demographic features. These features were concatenated (see region enclosed in the dashed box with the ECG features learned with the xResNet sans the last convolutional layer (figure 2). The joint features were then input into a fully connected layer and an activation layer with sigmoid activation to yield output probabilities of MI.

### Training process

For each deep learning model and each fold of cross-validations, recordings in the training set were cycled through 50 times (i.e. 50 epochs) with a batch size of 128. The final model was determined by comparing the validation loss from all training epochs and selecting the one that offered the smallest validation loss. The binary cross entropy was selected as the loss function, which measures the gap between estimated labels and true labels. All convolutional layer parameters were initialized based on the Xavier initialization method, which initializes weights with random values drawn from a uniform distribution. Our model training process did not rely on a rigid learning rate. Instead, it leveraged the scheme of cyclical learning rates which could reduce the guesswork for selecting an optimal learning rate and demonstrates improved classification accuracy (Smith 2015). We selected Adam as the optimizer due to its computational efficiency and low memory requirement.

The model training was performed on a computing server configured with a 24-core, 3.8 GHz CPU, 128 GB DDR4 RAM, and a GPU with 10 496 CUDA cores and 24 GB GDDR6X graphics memory. Key software and packages used to train the deep learning models included fast.ai (ver.1.0.61), PyTorch (ver.1.11.0), and Python (ver.3.10.7).

### Performance evaluation and model interpretability

Outputs of machine learning models after the sigmoid activation function were probabilities that span continuously in the range between zero and one. It required a preset probability threshold to generate estimated labels, which were compared with true labels to obtain true positive (TP), false positive (FP), true negative (TN), and false negative (FN). These were the building blocks to calculate traditional performance metrics, such as accuracy, sensitivity, specificity, precision, and F1 scores. We first used the area under the receiver operating characteristic curve (AUROC) as the main metric to compare the performance of different models, because it is threshold agnostic and can serve as an aggregated metric to evaluate the overall performance of machine learning models. We also plotted ROC curves from each CV step and their mean to visually compare the performance from the ECG-only and Multimodal models. In addition, we provided the five traditional metrics mentioned above by using the default probability threshold of 0.5 to further demonstrate the model performance. Given the unique class design in the study that incorporates four subclasses in the non-MI condition (i.e. NORM, STTC, HYP, and CD), an FP breakdown analysis was also performed to understand false positives rooted in different subclasses.

We examined the interpretability of the proposed Multimodal model by calculating the SHapley Additive exPlanations (SHAP) values of its input variables (i.e. age, sex, and ECG waveforms) (Lundberg and Lee 2017). SHAP is a method based on cooperative game theory that evaluates the importance of each feature contributing to the model output. We compared the maximal feature importance from each feature category to elucidate their levels of contribution to the classification of MI. Furthermore, feature importances across ECG tracings were evaluated to reveal regions of ECG beats to which the model paid heavy attention.

### Experiment #1 Impact of recording duration on model performance

We designed two experiments to investigate two separate important factors entrained in the development of machine learning models for classifying myocardial infarction. Experiment 1 concerns the duration lengths of ECG waveforms entering model training. Clinical standard 12-lead ECGs are typically presented in a 3 by 4 matrix of 2.5 s waveforms from each lead, sometimes accompanied by a 10 s rhythmic strip stacked at the bottom, despite digital recordings of full 10 s waveforms from all ECG leads ubiquitously captured in modern ECG devices. In Experiment 1, we compared the classification performance of ECG-only models trained from ECG recordings of 2.5 s, 5 s, 7.5 s, and 10 s, respectively. Such comparison helps answer the question of whether longer durations of ECG waveforms can help machines learn more information to improve the model performance.

### Experiment #2 Leveraging multiple modalities of data as model inputs

There is rich digital information routinely captured during intitial evaluation of MI, in addition to the standard ECG. Such information potentially provides more information about a patient’s condition than the ECG alone. In Experiment 2, we added patient demographic information (i.e. age and sex) as a first step into the multi-modality model design to obtain the Multimodal model and compare its classification performance with the ECG-only model. The comparison outcome elucidated whether leveraging additional clinical variables existing in the early clinical pipeline can enhance the classification power of machine learning models for detecting MI.

Given the small sample size of performance measures obtained in the study, the nonparametric two-sided Wilcoxon Rank Sum tests are adopted to compare the classification performance (in AUROC) of different models in these experiments. The significance level was set at 0.05, which is adjusted with Bonferroni correction for multiple comparisons.

## Results

### Longer durations offer improved performance

**Figure 3.**
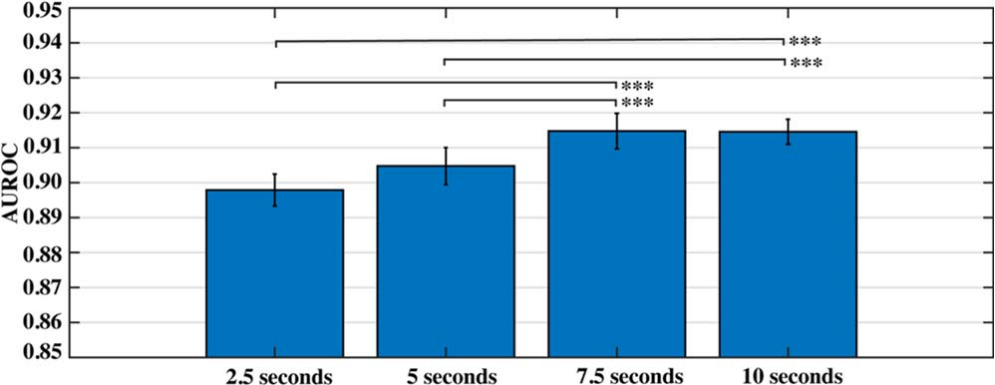
Performance comparison (in AUROC) between ECG-only models trained with different duration lengths of ECG waveforms (*** indicates *p* < *0.001*).

Figure 3 shows the performance in AUROC for ECG-only models trained with 2.5 s, 5 s, 7.5 s, and 10 s, respectively. It presents a generally increasing trend when a longer duration of ECG is provided for the model to learn. In the shorter duration group, the 2.5 s model achieved a mean AUROC of 89.8% (SD: 0.5%), which was not significantly different from the 5 s model (mean: 90.5%; SD: 0.5%). Similarly, in the longer duration group, the 7.5 s (mean AUROC: 91.5%; SD: 0.5%) and 10 s (mean AUROC: 91.5%; SD: 0.4%; 95% confidence interval: 91.2%–91.7%) models achieved comparable performance with no statistical significance. However, when comparing any models between the shorter (i.e. 2.5 s and 5 s) and longer duration groups (7.5 s and 10 s), a statistically significant difference can be observed (*p < 0.001*), with the longer duration models offering better performance compared to the shorter duration models.

### Single modality versus multiple modalities as inputs

**Figure 4.**
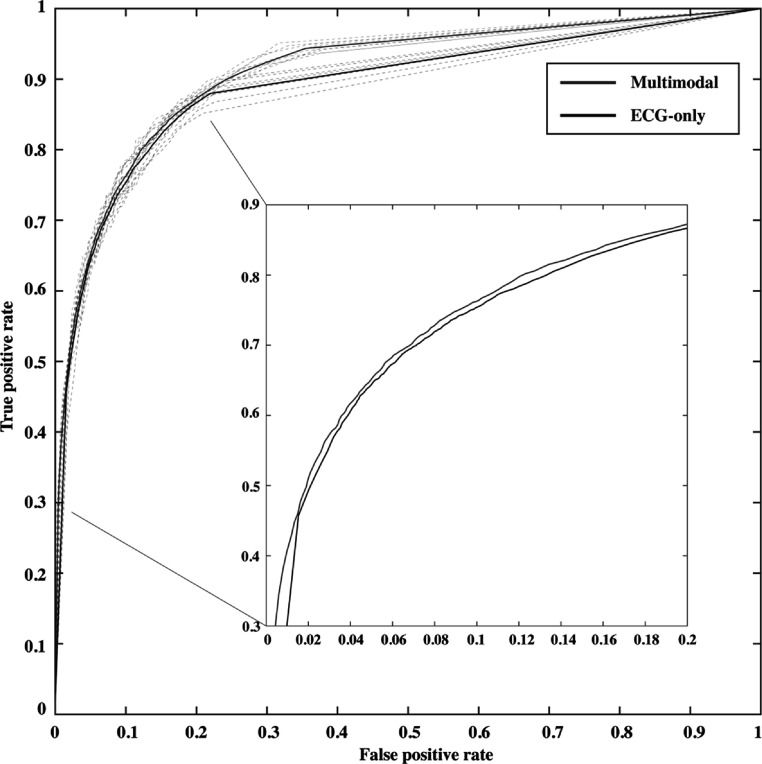
Comparison of the receiver operating characteristic (ROC) curves from the Multimodal and ECG-only models. Dashed lines represent ROC curves from each cross-validation step and solid lines represent the mean ROC curves.

Individual ROC curves from each CV fold are presented in figure 4, which shows superior performance from the Multimodal model over the ECG-only model. Tables 1 and 2 present a detailed review of the performance achieved by the ECG-only model and the Multimodal model, respectively. The ECG-only model was trained with 10 s ECG waveforms. The Multimodal model was trained with the combination of 10 s ECG and patient demographics. It can be observed that models obtained from different CV folds achieve stable performance with a small standard deviation in all performance metrics. It also shows that the Multimodal model improves metrics that reflect aggregated model performance, with increased AUROC (mean/SD: 92.1%/0.3%; 95% confidence interval: 91.9%–92.3%), accuracy (mean/SD: 87.4%/0.3%), precision (mean/SD: 77.9%%/1.2%), and F1 score (mean/SD: 73.5%/0.8%), compared to ECG-only models. Notably, the Multimodal model achieved comparable sensitivity (mean/SD: 69.7%/1.6%) to the 10 s ECG-only model, while offering improved specificity at 93.4% (SD: 0.6%). The two-sided Wilcoxon Rank Sum confirms the statistical significance in the AUROC achieved by the two models (*p < 0.001*).

**Table 1.** Classification performance of the ECG-only model trained with 10 s ECG.

CV Fold	AUROC	Accuracy	Sensitivity	Specificity	Precision	F1
1	0.919	0.872	0.685	0.934	0.777	0.728
2	0.918	0.877	0.712	0.932	0.777	0.743
3	0.915	0.872	0.694	0.932	0.774	0.732
4	0.909	0.868	0.682	0.930	0.766	0.722
5	0.909	0.866	0.700	0.921	0.749	0.723
6	0.917	0.872	0.705	0.928	0.768	0.735
7	0.916	0.867	0.709	0.921	0.750	0.729
8	0.914	0.877	0.725	0.928	0.771	0.747
9	0.914	0.871	0.682	0.934	0.776	0.726
Mean/SD	0.915/0.004	0.871/0.004	0.699/0.015	0.929/0.005	0.768/0.011	0.732/0.009

Abbreviations: CV = cross-validation; SD = standard deviation; AUROC = area under the receiver operating characteristic curve.

**Table 2.** Classification performance of the Multimodal model trained with a combination of 10 s ECG and patient demographic information.

CV Fold	AUROC	Accuracy	Sensitivity	Specificity	Precision	F1
1	0.923	0.873	0.673	0.941	0.791	0.727
2	0.919	0.875	0.684	0.939	0.789	0.733
3	0.925	0.879	0.723	0.932	0.780	0.750
4	0.917	0.871	0.694	0.930	0.770	0.730
5	0.918	0.872	0.682	0.935	0.779	0.727
6	0.922	0.874	0.714	0.927	0.767	0.74
7	0.921	0.875	0.691	0.937	0.786	0.735
8	0.920	0.870	0.707	0.924	0.758	0.732
9	0.922	0.879	0.703	0.938	0.791	0.744
Mean/SD	0.921/0.003	0.874/0.003	0.697/0.016	0.934/0.006	0.779/0.012	0.735/0.008

Abbreviations: CV = cross-validation; SD = standard deviation; AUROC = area under the receiver operating characteristic curve.

### Error analysis

**Figure 5.**
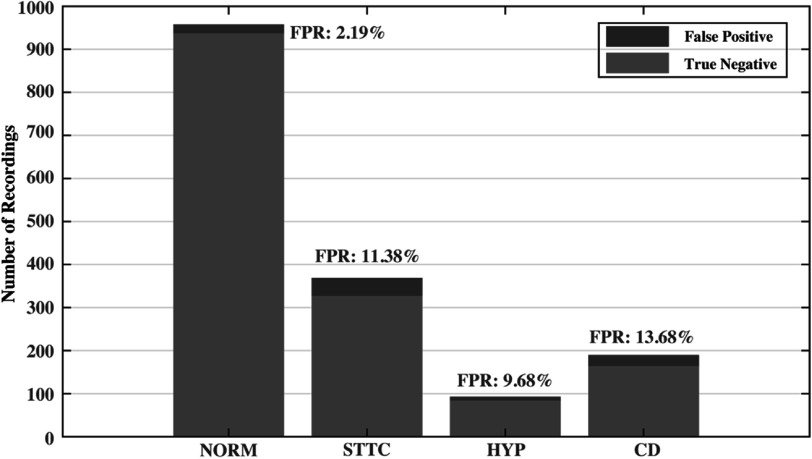
Breakdown of false positives across four subclasses in the non-MI condition. Abbreviations: FPR = false positive rate; NORM = normal ECG; STTC = ST/T change; HYP = hypertrophy; CD = conduction disturbance.

Figure 5 illustrates the breakdown of false positive classifications across four subclasses in the non-MI condition. We found significant variation in the proportions that different subclasses make up in the non-MI condition, but the model can correctly classify the majority of recordings in each subclass. When comparing the false positive rates (FPR) across different subclasses, it reveals that the model performs the best on ECG recordings with normal ECG beats (NORM) with the smallest FPR (2.19%), while MI-confounding subclasses (i.e. STTC, HYP, and CD) do challenge the performance of the classification model with much larger FPRs at 11.38%, 9.68%, and 13.68%, respectively.

### Model explainability

**Figure 6.**
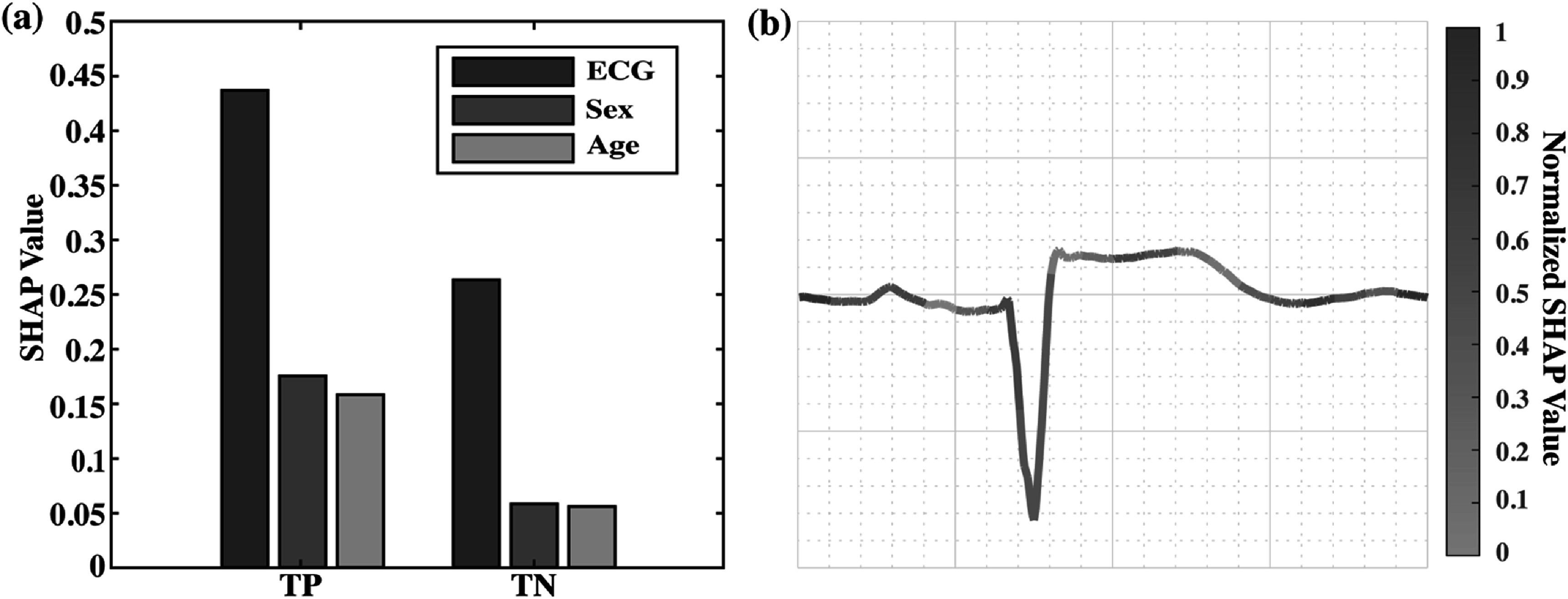
Feature importance (in SHAP value) in the Multimodal model. (a) Comparison of feature importance among three feature categories. (b) Feature importance in ECG tracings. The ECG in (b) is from a MI recording and the plot contains the first 5 s on Lead aVF.

Figure 6 presents the feature importance (in SHAP value) of different types of features used by the model, and the feature importance across time in the ECG tracing. Figure 6(a) reveals that the most important feature contributing to the model performance is ECG, followed by sex and age in MI recordings. The ranking stays true in control recordings but with reduced importance for all feature categories. Figure 6(b) presents feature importance averaged across all ECG beats in a MI recording on Lead aVF. For every 40 ms, the aggregated SHAP value was calculated and normalized to the range between 0 and 1 with respect to the whole ECG beat using the min-max normalization. It shows that the model’s attention distributes widely to different ECG landmarks. The model learned the presence of the Q wave and picked up the ST segment at J + 80. More importantly, the model also looked at the TP baseline, which might reflect the contextual information between the isoelectric level from the TP baseline and the ST segment, which is in concordance with the clinical guideline for identifying myocardial infarction (Thygesen *et al* 2018).

## Discussion

The key findings of this study are that the multimodal information helps improve the performance in AUROC of machine learning models for prompt identification of myocardial infarction and that the data-driven approach can leverage longer duration of ECG waveforms to make a better classification. Recognizing the underlying occlusion of coronary arteries early in time is associated with improved patient outcomes, as it helps reduce door-to-balloon time by, 1. optimizing EMS routes by sending MI patients directly to the nearest hospital with PCI-capability; 2. alerting the Cath Lab Team in advance allowing more time for the preparation of revascularization therapies. Reliable classification has potential to reduce inappropriate activation of the cardiac catheterization lab which saves the hospital operation cost and reserves limited clinical resources for patients in urgent need. With that in mind, our study purposefully constrained the types of data entered into model training to those commonly available during the initial care stage for MI. In addition, we dichotomized ECG labels into the MI class versus all other conditions, including both normal ECGs and chronic conditions that might confound the initial diagnosis of MI, such as LBBB and LVH. Such dichotomization reflects the clinical scenario for prompt screening MI. Under this direction, we investigated two important factors embroiled in the decision process of developing MI classification models using machine learning, i.e. data length and data types, which concern how much information and what information to enter model training, respectively.

Standard 12-lead ECGs are conventionally represented in the 2.5 s fashion as a convenient layout to provide an overview of information from all leads on one single screen. Healthcare providers are trained to make clinical inferences based on the standard 2.5 s layout. However, modern ECG machines store entire 10 s of ECG tracings. The longer duration of ECG may offer extra electrophysiological dynamics of hearts that help decypher the ongoing development of myocardial ischemia, given that acute MI is a rapidly developing condition. The longer data are more resilient to noise when a proportion of the recording is contaminated by artifacts. Machine learning by nature is a data-driven approach that can simultaneously navigate through information well-beyond human compacity, therefore may capitalize on the longer duration of data compared with the shorter one. This is demonstrated by results from Experiment 1 (figure 3) that a significantly improved classification performance can be obtained when using longer ECG waveforms (7.5 s and 10 s) than the shorter ones (2.5 s and 5 s) when trained with the same deep neural network.

Although standard 12-lead ECG remains the single most important clinical tool in the initial evaluation of myocardial infarction, mounting evidence suggests there is value in the integration of other information during the risk assessment process (Thygesen *et al* 2018). For example, studies show there is a sex difference in electrocardiographic manifestations of acute myocardial ischemia, with women presenting less ST elevation in ECG (Mauvais-Jarvis *et al* 2020). It also shows that there is a decrease in ST-elevation indicative of acute myocardial ischemia with increasing age (Thygesen *et al* 2018). Therefore, both age and sex have been incorporated into the latest universal definition of myocardial infarction (Thygesen *et al* 2018). In addition, patients with a previous diagnosis or at high risk of coronary artery disease may have altered ECG presentations (Delewi *et al* 2013). The inclusion of such information is therefore recommended by the international guideline to enhance the specificity of ECG findings (Thygesen *et al* 2018). In the present study, we designed a feature-infusion deep learning model that learns multimodal information including the patient demographics and physiologic information from ECG, and compared the obtained performance with the ECG-only model. Results from Experiment 2 (tables 1 and 2) show a significantly improved performance by the Multimodal model, demonstrating that the added patient demographics contribute to the model learning process by providing age and sex-adjusted discriminative features for the classification task.

Past studies have recognized the promise of machine learning in classifying myocardial infarction using ECG (Chang *et al* 2012, Sharma *et al* 2015, Reasat and Shahnaz 2017, Dohare *et al* 2018, Xiao *et al* 2018, Wang *et al* 2020, Strodthoff *et al* 2021). However, most of the existing studies formulated the classification problem (i.e. MI versus Normal) in the facilitation of identifying ECG features reflecting underlying ischemia at the expense of derailing from practical clinical applications, where both normal and other non-MI, even MI-confounding conditions exist besides MI. Our study is a big proponent of bearing this clinical need from the start, through the dichotomization of labels into MI and non-MI classes. Machine learning algorithms adopted in existing studies can be separated into shallow machine learning and deep learning. Our study followed the latter choice as we took advantage of the large public ECG dataset that provides ample recordings to train a sophisticated deep neural network (i.e. xResNet) (Wagner *et al* 2020). Comparison of performance across these studies is challenging due to the wide variety of experimental designs and performance metrics being measured. Here we selected accuracy as the metric to provide a rough estimate of where our model performance stands compared with others, as it is the most common metric reported across studies. Despite the inclusion of confounding conditions in the non-MI class that challenges the classification task in our study, the accuracy of 87.4% achieved by the Multimodal model is on par with other studies, which reported accuracies in the range of 82.5%~93.7% (Chang *et al* 2012, Sharma *et al* 2015, Reasat and Shahnaz 2017, Dohare *et al* 2018, Wang *et al* 2020, Strodthoff *et al* 2021). By excluding MI-confounding conditions during the evaluation of the Multimodal model on the test dataset, its accuracy can be raised to 90.5% (standard deviation: 0.6%). In addition, the ECG-only model trained with xResNet, the top-performing model in the benchmark study (Strodthoff *et al* 2021), also served as a state-of-the-art baseline model to fairly gauge the performance of the Multimodal model. The improvement in performance demonstrated the advantage of incorporating the additional data source in the classification task (see figure 4).

The aim of this study is to enhance the prompt detection of myocardial infarction by utilizing machine learning that is suitable for clinical use. It focuses on three key areas: creating classification labels that align with clinical practices, and determining the appropriate duration and type of information to use as input for the model. However, the study also has some limitations that require further research. One limitation is that the study prioritized the development of a practical ML model for MI detection, using an off-the-shelf model (xResNet), rather than introducing major algorithmic advancements. Further research is needed to design specialized ML models to enhance detection performance. Furthermore, the Multimodal model only included patient demographics as the additional data for model development, while other information, such as patient symptoms and past diagnoses, is also routinely collected during the initial care stage and has great potential to further elevate the classification performance. Our study is limited in that the dataset doesn’t contain the above information, but the unique network design of the proposed Multimodal model makes it readily expandable to other data modalities by simply adding new embedding modules. Therefore, a future study is warranted to investigate the value of those other data modalities. In addition, the FP breakdown analysis reveals a large difference in FPRs between the NORM subclass and MI-confounding subclasses. It is unclear whether it is due to the small number of recordings from those confounding subclasses that provide insufficient information for the model to learn, or the intrinsic challenge arising from the subtle features that distinguish these subclasses from the MI class. A further investigation by including additional recordings for these subclasses is needed for answering this question. Furthermore, the dataset adopted in the study was collected from multiple centers and thousands of patients, which offers a sample distribution with a strong estimation of the overall population. Nonetheless, the obtained model still requires extensive external validation to establish its generalizability, which is the key to successful model deployment.

## Data Availability

The data that support the findings of this study are openly available at the following URL/DOI: https://doi.org/10.13026/kfzx-aw45. Data will be available from 2 March 2022.
